# Prevalence and risk factors of retinopathy of prematurity in Iran: a systematic review and meta-analysis

**DOI:** 10.1186/s12886-018-0732-3

**Published:** 2018-04-02

**Authors:** Milad Azami, Zahra Jaafari, Shoboo Rahmati, Afsar Dastjani Farahani, Gholamreza Badfar

**Affiliations:** 1 Student Research Committee, Ilam University of Medical Sciences, Ilam, Iran; 2 Student Research Committee, Ilam University of Medical Sciences, Ilam, Iran; 3 Iranian National ROP Committee, Tehran, Iran; 4 Department of Pediatrics, Behbahan Faculty of Medical Sciences, Behbahan, Iran

**Keywords:** Meta-analysis, Retinopathy of prematurity, Iran, Prevalence, Risk factor

## Abstract

**Background:**

Retinopathy of prematurity (ROP) refers to the developmental disorder of the retina in premature infants and is one of the most serious and most dangerous complications in premature infants. The prevalence of ROP in Iran is different in various parts of Iran and its prevalence is reported to be 1–70% in different regions. This study aims to determine the prevalence and risk factors of ROP in Iran.

**Methods:**

This review article was conducted based on the preferred reporting items for systematic review and meta-analysis (PRISMA) protocols. To find literature about ROP in Iran, a comprehensive search was done using MeSH keywords in several online databases such as PubMed, Ovid, Science Direct, EMBASE, Web of Science, CINAHL, EBSCO, Magiran, Iranmedex, SID, Medlib, IranDoc, as well as the Google Scholar search engine until May 2017. Comprehensive Meta-analysis Software (CMA) Version 2 was used for data analysis.

**Results:**

According to 42 studies including 18,000 premature infants, the prevalence of ROP was reported to be 23.5% (95% CI: 20.4–26.8) in Iran. The prevalence of ROP stages 1, 2, 3, 4 and 5 was 7.9% (95% CI: 5.3–11.5), 9.7% (95% CI: 6.1–15.3), 2.8% (95% CI: 1.6–4.9), 2.9% (95% CI: 1.9–4.5) and 3.6% (95% CI: 2.4–5.2), respectively. The prevalence of ROP in Iranian girls and boys premature infants was 18.3% (95% CI: 12.8–25.4) and 18.9% (95% CI: 11.9–28.5), respectively. The lowest prevalence of ROP was in the West of Iran (12.3% [95% CI: 7.6–19.1]), while the highest prevalence was associated with the Center of Iran (24.9% [95% CI: 21.8–28.4]). The prevalence of ROP is increasing according to the year of study, and this relationship is not significant (*p* = 0.181). The significant risk factors for ROP were small gestational age (*p* < 0.001), low birth weight (*p* < 0.001), septicemia (*p* = 0.021), respiratory distress syndrome (*p* = 0.036), intraventricular hemorrhage (*p* = 0.005), continuous positive pressure ventilation (*p* = 0.023), saturation above 50% (*p* = 0.023), apnea (*p* = 0.002), frequency and duration of blood transfusion, oxygen therapy and phototherapy (*p* < 0.05), whereas pre-eclampsia decreased the prevalence of ROP (*p* = 0.014).

**Conclusion:**

Considering the high prevalence of ROP in Iran, screening and close supervision by experienced ophthalmologists to diagnose and treat the common complications of pre-maturity and prevent visual impairment or blindness is necessary.

## Background

Retinopathy of prematurity (ROP) refers to the developmental disorder of the retina in premature infants and is one of the most serious and most dangerous complications in premature infants.

Embryonic retinal arteries start to grow in the third month of pregnancy and their development ends at birth. Therefore, the stages of evolution of the eye are defective in premature infants, and the growth of the vessels is either stopped or unusual, and ultimately, the vessels become very fragile, which can lead to visual impairment in severe cases [1].

Despite considerable progress made in the treatment of ROP, it is still a common cause of reduced vision in children in developed countries, and its prevalence is increasing [2–4]. This is a preventable disease and responds to treatments appropriately if diagnosed at early stages, but in case of delayed diagnosis and treatment, it may lead to blindness [5].

The first incidences of ROP were reported in the 1940s and 1950s, mainly as a result of the use of supplemental oxygen without supervision (first epidemic). Although the survival of premature infants improved in the following decades, and despite improved monitoring methods for oxygen supplements, ROP emerged with an increasing incidence (second epidemic) [6]. Over the past decade, the increasing incidence of ROP blindness has been recorded in low-income countries. Studies show that ROP is the leading cause of blindness in China, Southeast Asia, South America, Latin America, and Eastern Europe, especially in urban centers of newly industrialized countries, and this is referred to as the “third epidemic” [7].

ROP is a multifactorial disease and the most important risk factors are preterm delivery, especially before the 32nd week of gestation and birth weight less than 1500 g. Apnea, intraventricular hemorrhage, various maternal factors (diabetes, preeclampsia, mother’s smoking), respiratory disorders, infection, vitamin E deficiency, heart disease, increased blood carbon dioxide, increased oxygen (O_2_) consumption, decreased PH, decreased blood O2, bradycardia, transfusion, amount of received oxygen and duration of ventilation are other risk factors for ROP [8–10].

The prevalence of ROP in different regions of Iran is different and its prevalence is reported to be 1–70% in different regions [11–14]. Considering the abovementioned issues and the importance of the subject, as well as the diversity of reports in Iranian studies, it is necessary to carry out more extensive and precise studies. Meta-analysis is a method that collects and analyzes multiple research data with a common purpose to provide a reliable estimate of the impact of some interventions or observations in medicine [15, 16]. Obviously, the sample size in meta-analysis becomes larger by collecting data from several studies and therefore the range of changes and probabilities will be reduced; therefore, the significance of statistical results increases [16, 17]. This study aims to determine the incidence and risk factors for ROP in Iran.

## Methods

### Study protocol

This review article was conducted based on the preferred reporting items for systematic review and meta-analysis (PRISMA) protocols [16]. The study was conducted in five stages: design and search strategy, a collection of articles and their systematic review, evaluation of inclusion and exclusion criteria, qualitative evaluation and statistical analysis of data. To avoid bias in the study, each of the above steps was carried out by two researchers independently. In case of differences in the results obtained by the two researchers, a third researcher intervened to reach an agreement.

### Search strategy

To find literature about ROP in Iran, a comprehensive search was done using the terms (Retinopathy of Prematurity [MeSH]) AND (“Incidence” [MeSH] OR “Epidemiology” [MeSH]), OR (“Prevalence” [MeSH]) AND (“Iran” [MeSH]) in 7 international databases including PubMed, Ovid, Science Direct, EMBASE, Web of Science, CINAHL, EBSCO, and 5 national databases including Magiran, Iranmedex, SID, Medlib, IranDoc, as well as Google Scholar search engine until May 2017. References to all relevant articles were reviewed. Due to the inability of Iranian databases to search using Boolean operators (AND, OR and NOT), searches on these databases were only performed using the keywords.

### Inclusion and exclusion criteria

Articles with the following characteristics were chosen for meta-analysis: 1. Original research papers published either in Persian or English; 2. Medical dissertations; 3. Review of the prevalence or risk factors for ROP. The exclusion criteria were: 1. Non-random sample for estimating the prevalence; 2. Being irrelevant to the topic; 3. Congress papers; 4. Sample size other than premature infants; 5. Non-Iranian studies; 6. Review articles, case reports, editorials; 7. Duplicate studies and 8. Low-quality studies.

### ROP detection criteria

ROP was diagnosed by an expert through examination of retinas of infants using indirect ophthalmoscope.

### Selection of studies

First, all related articles (articles with affiliations containing Iranian authors) were collected and a list of titles was prepared at the end of the search and removal of duplicates. After blinding the specifications of the articles by on researcher (Milad Azami), including the name of the journal and the name of the author, the full text of the articles was presented to the researchers. Each article was studied by two researchers independently (Gholamreza Badfar, Afsar Dastjani Farahani). If the article was rejected, the reason for this rejection was mentioned. In case of disagreement between the two authors, the article was judged by the team of researchers.

### Quality of studies

Using the standard modified Newcastle Ottawa Scale (NOS) checklist [18], which included 8 sections. Thus, the minimum and maximum score available on this checklist were 0 and 8, respectively. Accordingly, the studies were divided into three categories: 1. low quality with a score less than 5; 2. moderate quality with a score of 5–6; and 3. high quality with a score of 7–8. Finally, the moderate to high quality studies were selected for the meta-analysis stage.

### Data extraction

The raw data of the prepared articles were extracted using a premade checklist. The checklist includes the name of the authors, published year the year of study, the location of the study, the study design, quality score, sample size, the prevalence of ROP, the ROP detection criteria, the prevalence of ROP based on gender (ROP) and ROP risk factors.

### Statistical analysis

In each study, the prevalence of ROP was considered as the probability of binomial distribution. To evaluate the heterogeneity of the studies, Cochran’s Q test and I^2^ index were used [19]. There are three categories for the I^2^ index: heterogeneity lower than 25%, heterogeneity between 25% and 75% and heterogeneity more than 75%. Considering the heterogeneity of the studies, a random effects model was used to combine ROP prevalence. For ROP risk factors, the fixed effects model and the random effects model were used, respectively in the case of low heterogeneity and high heterogeneity in the meta-analysis [20, 21]. Sensitivity analysis was performed to identify the influence of a single study on the combined result incidence or any risk factors (with ≥ 7 studies). In order to identify the cause of heterogeneity of ROP prevalence, sub-groups analysis of ROP were carried out based on geographical region, province and quality of studies, while the meta-regression model (method of moments) was carried out based on the year of studies [22]. Egger and Begg’s tests were used to identify publications bias. Data analysis was performed using Comprehensive Meta-Analysis Software Version 2 and the significance level in the tests was considered to be lower than 0.05.

## Results

### Search results and characteristics

**Fig. 1:**
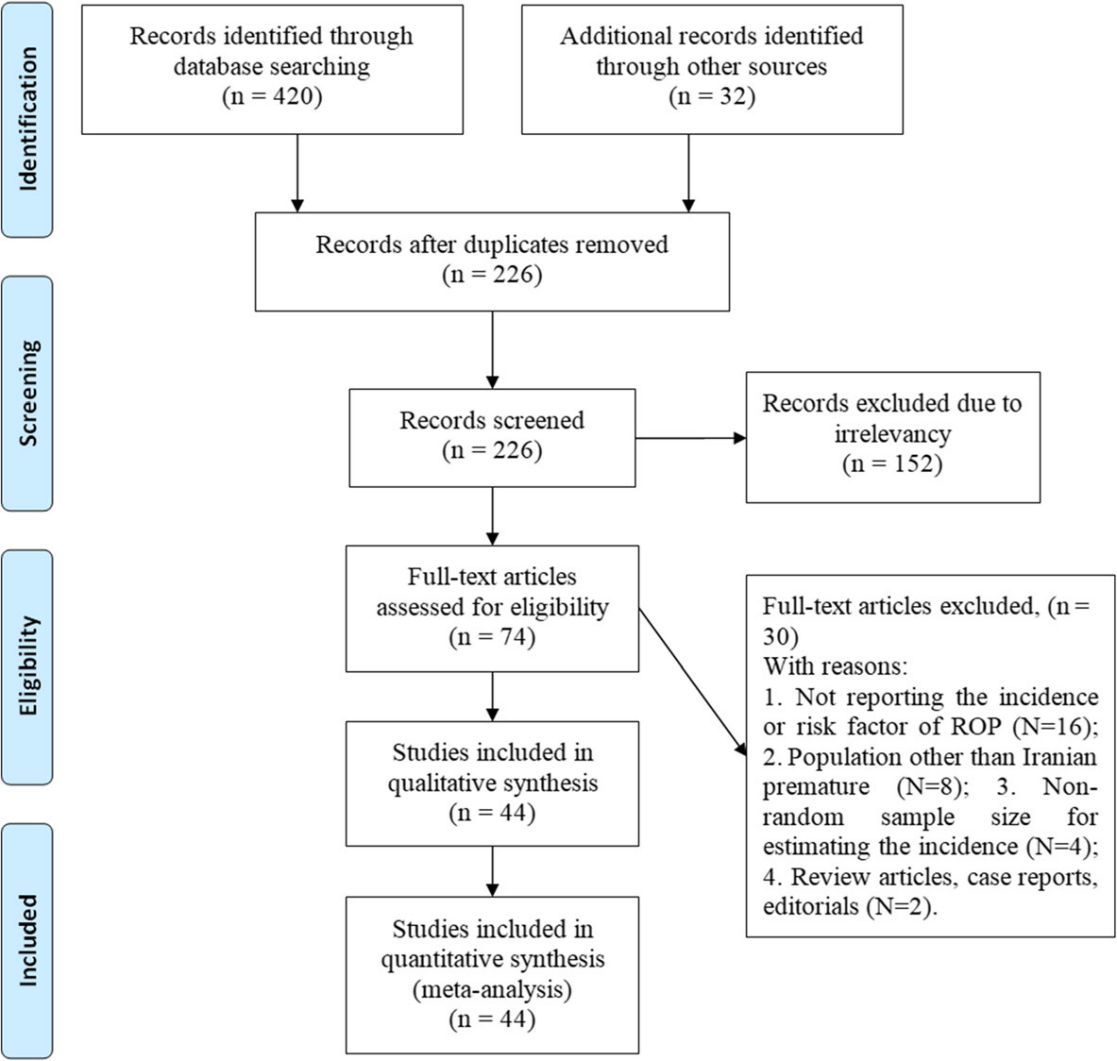
PRISMA flowchart for the selection of studies

In the initial search, 452 studies were found to be related to the topic. Two independent researchers reviewed the title and the abstract. If the title or abstract was likely to be related to the topic, the full text was reviewed. After reviewing the full text of 74 relevant articles, 30 articles were omitted due to lacking the necessary criteria and finally 44 qualified studies entered the qualitative assessment stage (Fig. 1). Table 1 shows the characteristics of each study.

**Table 1:** Summary of demographic characteristics in studies into a meta-analysis

Ref.	First author, Published Year	Year of study	GA^a^ (week)	BW^b^ (gr)	Place	Sample size			Prevalence (%)	Quality
						All	Non-ROP^c^	ROP		
[11]	Naderian Gh, 2011	2009	< 34	And ≤ 1800	Isfahan	100	71	29	29	Moderate
[11]	Naderian Gh(1), 2011	2009	< 34	And ≤ 1800	Isfahan	100	58	42	42	Moderate
[12, 13]	Mostafa Gharebagh M, 2012	2008	< 34	–	Tabriz	71	41	30		High
[14]	Nakhshab M, 2016	2014	< 30 or < 34^d^	–	Sari	146	122	24	16.44	High
[52]	Naderian G, 2009	2002	25–34	And 600–1800	Isfahan	796	662	134	16.8	Moderate
[53]	Hosseini H, 2009	2006	< 34	–	Shiraz	1024	1004	20	1.95	High
[54]	Karkhaneh R, 2005	2000	≤ 37	And ≤ 2500	Tehran	185	162	23	12.4	High
[55]	Naderian G, 2010	2003	–	–	Isfahan	604	498	106	17.5	High
[56]	Mansouri M, 2007	2004	≤ 32	And ≤ 1500	Tehran	147	103	44	29.9	High
[57]	Nakshab M, 2003	2001	–	≤ 2500	Sari	68	60	8	11.7	High
[58]	Daraie G, 2016	2008	< 37	Or < 2000	Semnan	270	267	3	1.1	Moderate
[59]	Fayazi A,2009	2005	< 32	Or < 1500 or 1500–2500^*^	Tabriz	399	370	29	7.26	Moderate
[60]	Sadeghi K, 2008	2006	< 36	And < 2000	Tabriz	150	124	26	17.3	Moderate
[61]	Ebrahimiadib N, 2016	2011	< 37	Or < 3000	Tehran	1896	1326	570	30.06	Moderate
[62]	Ghaseminejad A, 2011	2006	≤ 36	And ≤ 2500	Kerman	83	59	24	29	High
[63]	Khatami F, 2008	2000	< 34	Or < 2000	Mashhad	50	36	14	28	Moderate
[64]	Sabzehei MK, 2013	2007	–	< 1500	Tehran	414	343	71	17.14	Moderate
[65]	Saeidi R, 2009	2005	≤ 32	Or < 1500	Mashhad	47	43	4	8.5	Moderate
[66]	Azin Far B, 2005	2001	< 29	And < 1500	Babol	100	56	44	44	High
[67]	Karkhanehyousefi N, 2009	2009	–	–	Babol	100	61	39	39	Moderate
[68]	Ebrahimzadeh A, 2009	2003	–	–	Tehran	1343	874	469	34.9	High
[69]	Mirzaee SA, 2010	2008	–	< 2000	Tehran	74	50	24	324	Moderate
[70]	Mousavi Z, 2009	2001	24–36	And 600–2900	Tehran	797	540	257	32.24	Moderate
[71]	Fouladinejad M, 2009	2004	≤ 34	–	Gorgan	89	84	5	5.6	High
[72]	Mousavi S, 2008	2001	24–36	And 600–2800	Tehran	693	474	219	31.6	Moderate
[73]	Sadeghzadeh M, 2016	2001	–	450–3000	Zanjan	78	77	1	1.2	Moderate
[74]	Bayat-Mokhtari M, 2010	2006	–	< 1500 Or 1500–2000*	Shiraz	199	115	84	42	High
[75]	Karkhaneh R, 2001	1997	< 37	Or < 2500	Tehran	150	141	9	6	High
[76]	Babaei H, 2012	2009	–	≤ 1500	Kermanshah	84	73	11	13.1	Moderate
[77]	Abrishami M, 2013	2006	< 32	–	Mashhad	122	90	32	26.2	High
[78]	Riazi-Esfahani M, 2008	2002	≤ 37	And ≤ 2500	Tehran	165	125	40	24.24	Moderate
[79]	Alizadeh Y, 2015	2005	≤ 36	And ≤ 2500	Rasht	310	246	64	20.6	High
[80]	Mousavi SZ, 2010	2003	–	–	Tehran	605	415	190	31.4	Moderate
[81]	Mousavi Z, 2010	2003	–	–	Tehran	1053	673	380	36.1	High
[82]	Feghhi M, 2012	2006	< 32	And ≤ 2000	Ahvaz	576	393	183	32	High
[83]	Afarid M, 2012	2006	≤ 32	And ≤ 2000	Shiraz	787	494	293	37.2	Moderate
[84]	Ahmadpourkacho M, 2014	2009	< 28	And < 1500 or 1500–2000*	Babol	256	76	180	70.31	High
[85]	AhmadpourKacho M, 2014	2007	< 34	And < 2000	Babol	155	85	70	45.2	Moderate
[86]	Rasoulinejad SA, 2016	2007	< 36	And < 2500	Babol	680	374	306	45	High
[87]	Karkhaneh R, 2008	2003	< 37	–	Tehran	953	624	329	34.5	High
[88]	Khalesi N, 2015	2013	–	–	Tehran	120	60	60		Moderate
[89]	Ebrahim M, 2010	2004	< 37	–	Babol	173	140	33	19.1	High
[90]	Roohipoor R, 2016	2012	≤ 37	And ≤ 3000	Tehran	1932	1362	570	3	High
[91]	Mansouri M, 2016	2013	< 34	Or < 2000	Sanandaj	47	42	5	10.6	High

^a^Gestational age; ^b^Birth weight; ^c^Retinopathy of prematurity; ^d^With unstable condition

### Prevalence

**Fig. 2:**
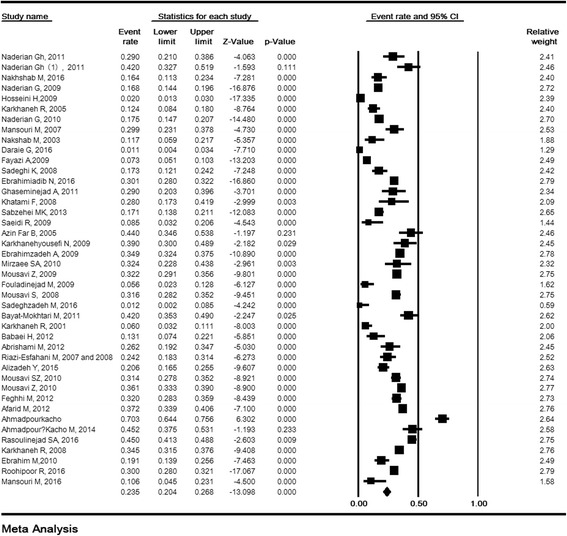
The prevalence of retinopathy of prematurity in Iran. Random effects model

Reviewing 42 studies with a total sample size of 18,000 premature infants, the prevalence of ROP in Iran was estimated to be 23.5% (95% CI: 20.4–26.8). The lowest and highest prevalence was related to the studies in Semnan (2008) (1.1%) (58) and in Babol (2009) (70.3%) (84), respectively (Fig. 2).

### Sensitivity analysis and cumulative analysis for ROP

**Fig. 3:**
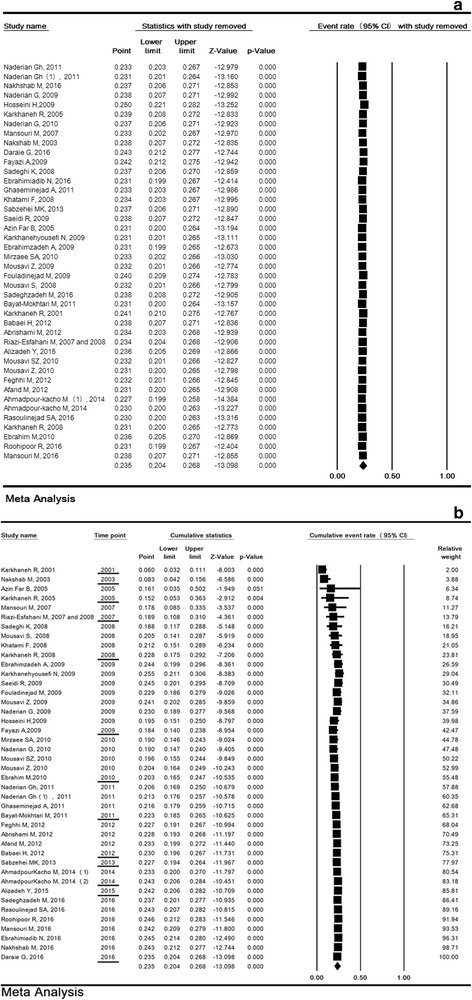
Sensitivity analysis (**a**) and cumulative analysis based on the year of publication (**b**) for prevalence of retinopathy of prematurity in Iran. Random effects model

The sensitivity analysis of the prevalence or risk factors of ROP and its 95% confidence interval (CI) was estimated simultaneously regardless of one study and the results showed that the incidence or risk factors of ROP were not significantly changed before and after the deletion of each study. (Fig. 3a). Cumulative analysis for incidence of ROP based on the year of publication is shown in Fig. 3b.

### Subgroup analysis of ROP prevalence based on geographic region

In the reviewed studies, 2, 4, 12, 4, and 20 studies were related to the West, East, North, South, and Center of Iran, respectively. The prevalence of ROP in the five regions of Iran is shown in Table 2 and the lowest incidence of ROP was in west of Iran (12.3% [95% CI: 7.6–19.1]), while the highest prevalence was related to the center of Iran (24.9% [95% CI: 21.8–28.4]) (Table 2).

**Table 2:** The prevalence of ROP based on region, gender, provinces and quality of studies

Variable		Studies (N^a^)	Sample (N)	Heterogeneity		95% CI^b^	Prevalence (%)
				I^2^	*P*-Value		
Region	Center	20	12,355	93.65	< 0.001	21.8 to 28.4	24.9
	East	4	302	57.79	0.07	17 to 33	24.1
	North	12	2626	97.09	< 0.001	15.9 to 37.1	25
	South	4	2586	98.60	< 0.001	9.2 to 37.1	20.5
	West	2	131	0	0.67	7.6 to 19.1	12.3
	Test for subgroup differences: Q = 9.67, df(Q) = 4, *P* = 0.046						
Gender	Boys	11	1467	92.65	< 0.001	11.9 to 28.5	18.9
	Girls	11	1184	85.02	< 0.001	12.8 to 25.4	18.3
	Rate ratio of boys to girls: OR^c^ = 1.07(0.86 to 1.33, *P* = 0.501)						
Provinces	Khozestan	1	576	0	–	28.3 to 35.9	32
	Mazandaran	8	1678	95.77	< 0.001	23.5 to 48.2	34.8
	Isfahan	4	1600	92.48	< 0.001	16.5 to 35	24.6
	Golestan	1	89	0	–	2.3 to 12.8	5.6
	Kerman	1	83	0	–	20.3 to 39.6	29
	Kermanshah	3	84	0	–	7.4 to 22.1	13.1
	Razavi Khorasan	3	219	67.89	0.044	12.4 to 34.2	21.3
	Guilan	1	310	0	–	16.5 to 25.5	20.9
	Kurdistan	1	47	0	–	4.5 to 23.1	10.6
	Semnan	1	270	0	–	0.4 to 3.4	1.1
	Fars	3	2010	99.09	< 0.001	4 to 50.8	17.2
	East Azarbaijan	2	549	91.32	0.001	4.6 to 25	11.3
	Tehran	14	10,407	91.32	< 0.001	25.1 to 31	28
	Zanjan	1	78	0	–	0.2 to 8.5	1.2
	Test for subgroup differences: Q = 97.59, df(Q) = 13, *P* < 0.001						
Quality	Medium	20	7760	63.68	< 0.001	16.6 to 28.0	23.5
	High	22	10,240	96.65	< 0.001	19.1 to 28.7	23.5
	Test for subgroup differences: Q = 0, df(Q) = 1, *P* = 0.995						

^a^Number

^b^Confidence interval

### Subgroup analysis of ROP prevalence based on province

**Fig. 4:**
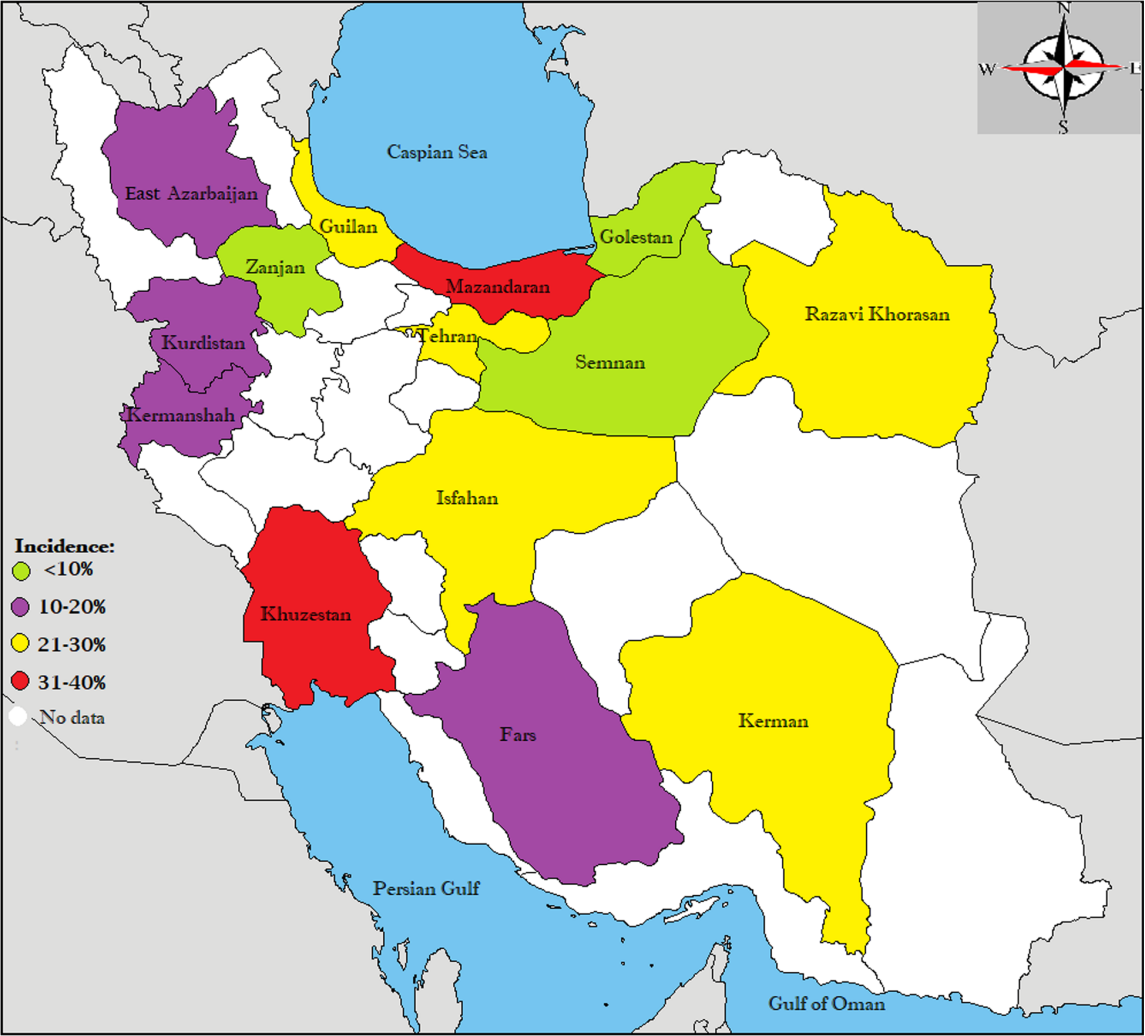
Geographical distribution of retinopathy of prematurity in Iran

Table 2 and Fig. 4 show the prevalence of ROP based on Iran’s provinces. The highest prevalence was in provinces of Mazandaran (34.8%) and Khuzestan (32%), and the lowest prevalence was in the provinces of Semnan (1.1%) and Zanjan (1.2%).

### Subgroup analysis of ROP prevalence based on the quality of studies

The prevalence of ROP in moderate and high-quality studies was 23.5% (95% CI: 16.6–28.0) and 23.5% (95% CI: 19.1–28.7), respectively, and the difference was not statistically significant (*p* = 0.995) (Table 2).

### The prevalence of ROP based on gender

The prevalence of ROP in girls and boys premature infants was 18.3% (95% CI: 12.8–25.4) and 18.9% (95% CI: 11.9–28.5), respectively. Their difference was not statistically significant (*P* = 0.501) (Table 2).

### The prevalence of ROP based on stage

**Fig. 5:**
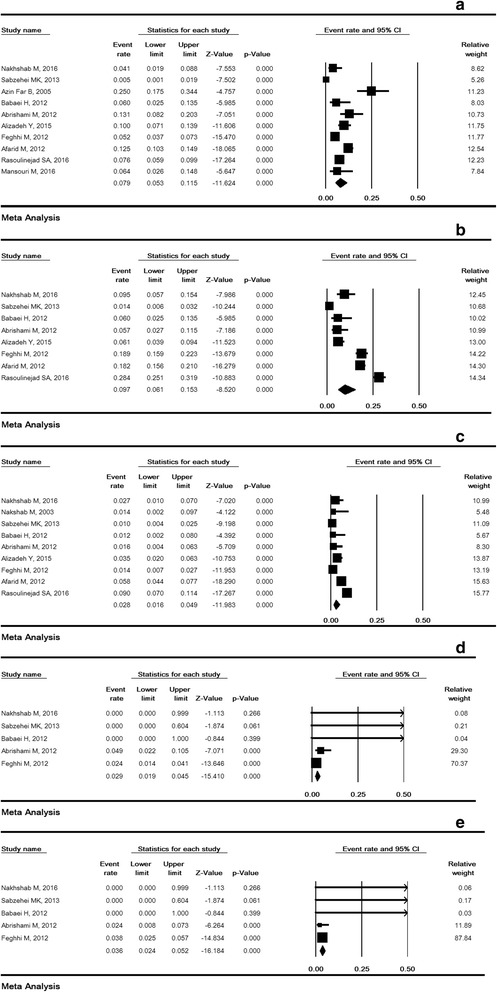
The prevalence of stages I (**a**), II (**b**), III (**c**), IV (**d**), V (**e**) retinopathy of prematurity. Random effects model

The prevalence of stages 1, 2, 3, 4 and 5 were reported in 10, eight, nine, five, and five studies, respectively. Fig. 5 shows the prevalence of ROP at different stages. The prevalence of stages 1, 2, 3, 4 and 5 was 7.9% (95% CI: 5.3–11.5), 9.7% (95% CI: 6.1–15.3), 2.8% (95% CI: 1.6–4.9), 2.9% (95% CI: 1.9–4.5), and 3.6% (95% CI: 2.4–5.2), respectively.

### Meta-regression

**Fig. 6:**
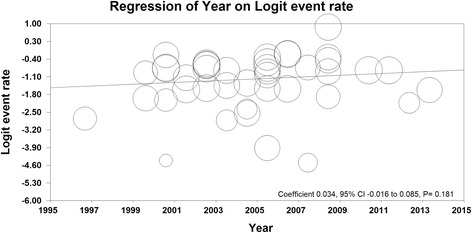
Meta-regression of ROP prevalence based on years of studies

Meta-regression model in Fig. 6 shows that the incidence of ROP is increasing according to the year of study, and this relationship is not statistically significant (meta-regression coefficient: 0.034, 95% CI -0.016 to 0.085, *P* = 0.181).

### Publication bias

**Fig. 7:**
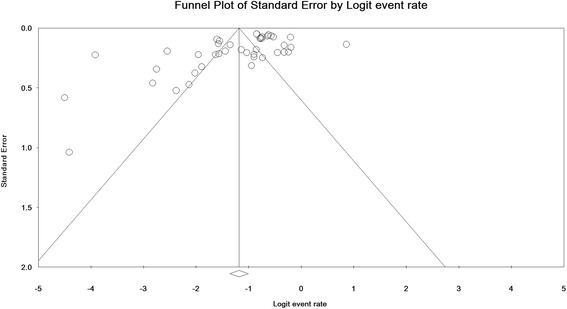
Publication bias in the studies

The significance level of publication bias in the reviewed studies was 0.003 and 0.002 according to Egger and Begg’s tests, respectively, which is shown in Fig. 7.

### ROP risk factors

The meta-analysis results of evaluating the risk factors of ROP are shown in Table 3. ROP risk factors include certain variables such as continuous positive pressure (CPAP) (*P* = 0.023), the prevalence of blood transfusion (*P* = 0.001), septicemia (*P* = 0.021), weight < 1000 g (*P* < 0.001), weight <  1500 g (*P* < 0.0001), frequency of phototherapy (*P* < 0.0001), the frequency of oxygen therapy (*P* = 0.049), apnea (*P* = 00.2), intraventricular hemorrhage (IVH) (*P* = 0.005), respiratory distress syndrome (RDS) (*P* = 0.036), gestational age (GA) ≤ 28 W(week) (*P* < 0.001), GA ≤32 W (*P* < 0.001), saturation over 50% (*P* < 0.001), mean GA (*P* < 0.001), mean weight (*P* < 0.0001), oxygen therapy duration (*P* < 0.001) and phototherapy duration (*P* < 0.0001); however, preeclampsia significantly decreases the prevalence of ROP (*P* = 0.014).

**Table 3:** Risk factor for retinopathy of prematurity in Iran

Variables	Studies(*N*^a^)	Sample (*N*)		Heterogeneity		OR (95%CI^b^)	*P*-Value	Model in Meta-analysis
		Case	Control	I^2^	*P*-Value			
Twin birth	4	804	1868	46.97	0.129	1.62 (0.94 to 2.81)	0.081	Random^c^
Mechanical ventilation	6	1131	2493	73.35	0.002	1.81 (0.80 to 1.73)	0.39	Random
Continuous positive pressure ventilation	2	62	131	64.11	0.095	3.97 (1.21 to 13.01)	0.023	Random
Blood transfusion (N)	16	1820	4167	91.34	< 0.001	2.38 (1.43 to 3.94)	0.001	Random
Septicemia	11	1327	2965	80.75	< 0.001	1.96 (1.10 to 3.48)	0.021	Random
Birth weight < 1000 g	9	573	2093	59.65	0.011	4.16 (2.35 to 7.35)	< 0.001	Random
Birth weight < 1500 g	10	559	1984	43.34	0.069	3.74 (2.54 to 5.49)	< 0.001	Random
Phototherapy (N)	11	1380	3355	80.69	< 0.001	1.50 (1.00 to 2.27)	0.049	Random
Oxygen therapy (N)	14	726	3124	87.39	< 0.001	3.06 (1.29 to 7.27)	0.011	Random
Need for resuscitation	2	56	212	86.50	0.006	5.01 (0.18 to 135.71)	0.338	Random
Apnea	3	114	492	72.08	0.028	4.41 (1.70 to 11.40)	0.002	Random
Congenital heart disease	2	50	246	67.29	0.08	2.13 (0.10 to 45.62)	0.626	Random
Inter-ventricular hemorrhage	11	1223	3178	76.36	< 0.001	2.24 (1.2 to 3.95)	0.005	Random
Acidosis	3	132	296	62.62	0.069	2.56 (0.81 to 8.06)	0.106	Random
Cesarean section	4	375	830	47.88	0.124	1.08 (0.53 to 2.18)	0.82	Random
Preeclampsia	2	108	237	0	0.82	0.12 (0.02 to 0.65)	0.014	Fixed^d^
Respiratory distress syndrome	11	2039	2618	80.13	< 0.001	1.64 (1.03 to 2.61)	0.036	Random
Saturation above 50%	4	118	656	30.30	0.23	8.35 (3.14 to 22.18)	< 0.001	Random
Normal Vaginal Delivery	4	375	830	46.63	0.132	1.01 (0.50 to 2.02)	0.969	Random
Multiple pregnancy	6	1199	2518	40.20	0.137	0.92 (0.73 to 1.16)	0.517	Random
Gestational age ≤ 28	6	551	1440	75.88	< 0.001	5.20 (2.31 to 11.73)	< 0.001	Random
Gestational age ≤ 32	9	689	1885	64.84	0.004	7.88 (4.62 to 13.46)	< 0.001	Random
Birth weight (gr)	7	1495	2893	97.30	< 0.001	0.98 (0.97 to 0.99)	< 0.001	Random
Gestational age (week)	7	1495	2893	84.20	< 0.001	0.67 (0.59 to 0.770)	< 0.001	Random
Variables	Studies(*N*^a^)	Sample (*N*)		Heterogeneity		Mean Difference (95% CI^b^)	*P*-Value	
		Case	Control	I^2^	*P*-Value			
Gestational age (weeks)	18	1835	4126	94.53	< 0.001	2.08(1.50 to 2.66)	< 0.001	Random
Birth weight (gr)	19	1782	4519	95.94	< 0.001	305.39(236.09 to 374.69)	< 0.001	Random
Oxygen therapy (day)	11	1399	3214	96.04	< 0.001	−4.36(−6.09 to −2.63)	< 0.001	Random
Phototherapy (days)	4	78	308	83.80	< 0.001	−2.08(−3.81 to −0.35)	< 0.001	Random
Apgar score in the first minute	3	174	216	63.30	0.66	1.07(0.45 to 1.68)	0.001	Random
Apgar score	3	64	272	76.34	0.015	0.43(−0.25 to −1.13)	0.21	Random
Mechanical ventilation (days)	2	114	154	88.81	0.003	−4.53(−9.17 to 0.10)	0.55	Random
Bilirubin (mg/di)	3	54	186	7.70	0.33	−0.27(−1.40 to 0.86)	0.63	Random
Blood transfusion (duration)	2	98	151	0	0.98	−0.69(−0.96 to − 0.42)	< 0.001	Fixed
clinical risk index for babies	2	161	250	58.84	0.11	−0.62(− 1.40 to 0.16)	0.11	Random

^a^Number

^b^Confidence interval

^c^Random effects model

^d^Fixed effects model

## Discussion

The present study is the first systematic and meta-analytic review on the prevalence and risk factors of ROP in Iran. The results of this meta-analysis showed that the prevalence of ROP in 18,000 Iranian premature infants was 23.5%, and the prevalence for stages 1, 2, 3, 4 and 5 was 7.9%, 9.7%, 2.8%, 2.9% and 3.6%, respectively. In this study, the level of heterogeneity was high for ROP studies (95.6%). The results of the subgroup analysis showed that geographic regions and the provinces could be a cause of high heterogeneity. However, this difference can be a reflection of studies conducted on different samples based on the GA or neonatal weight.

ROP is still a major cause of potentially preventable blindness around the world [23]. According to guidelines published by the American Academy of Ophthalmology, the American Academy of Children, and the American Association for Ophthalmology for Children and Strabismus for ROP screening, infants weighing less than 1500 g or GA ≤ 30 weeks, and infants weighing between 1500 and 2000 g or GA > 30 weeks with an unstable clinical course should receive dilated ophthalmoscopy examinations for ROP [24].

The prevalence of ROP in various studies is mainly due to differences in mean GA and birth weight of infants in each study. Based on GA, the prevalence of ROP significantly decreases from 77.9% in GA 24–25 to 1.1% in GA 30–31, which indicates the direct role of GA in ROP incidence. These results are completely consistent with the data published in other literature [25–31]. Moreover, in a meta-analysis study in Iran, the prevalence of prematurity was reported to be 9.2% (95% CI: 7.6–10.7) [32]. Therefore, the high prevalence of ROP in Iran (23.5%) can be explained by the high prevalence of prematurity.

In a study by Tabarez-Carvajal et al. among 3018 premature infants, the incidence of stages 1, 2, 3, 4, and 5 was reported to be 8.34%, 8.78%, 1.9%, 0.03%, and 0.30%, respectively [33]. In another study by Abdel HA et al., the prevalence of ROP stage 1 was 10.4%, stage 2 was 5.2% and stage 3 was 3.45%, and none of the infants had ROP at stages 4 or 5 [34]. But in the present study, the prevalence of ROP stages 4 and 5 was higher.

ROP is a multi-factorial disease, and in the present study, the strongest risk factor for ROP was prematurity and low birth weight. Most studies have demonstrated that prematurity and low birth weight are the strongest predictive factors of ROP, which indicates the crucial role of factors associated with the progression of the ROP disease [35–45].

After low birth weight and prematurity, exposure to oxygen for a long period and saturation over 50% were the most important risk factors for ROP in this study, which was consistent with the results of many other studies [42–47]. Due to inadequate antioxidant defense system, premature infants are not evolved to live in an oxygen-rich ectopic environment [48, 49]. Oxidative stress is the result of various organs’ exposure to free radicals of oxygen after being exposed to high concentrations of oxygen, which can lead to the progression of many pathogens such as ROP, necrotizing enterocolitis, IVH, bronchopulmonary dysplasia, and periventricular leukomalacia [50, 51].

In this study, other significant relationships with ROP were also found, including frequency and duration of blood transfusion, phototherapy, septicemia, apnea, IVH, and RDS. The comparison between the risk factors in our study and other reports is shown in Table 4.

**Table 4:** Risk factor for retinopathy of prematurity in other studies

Study details	GA (weeks)	BW (gr)	Risk factors
Reyes et al., 2017. Oman [46]	< 32	< 1500	low BW, low GA, duration of invasive ventilation, duration of oxygen therapy, duration of nasal CPAP, late onset clinical or proven sepsis
Shah et al., 2005 Singapore [40]	< 32	< 1500	Preeclampsia, low BW, prolonged duration of ventilation, pulmonary hemorrhage and CPAP
Yau et al., 2016, China [45]	< 32 and > 32	< 1500	low GA, low BW, preeclampsia, gestational diabetes mellitus, inotrope use, postnatal hypotension, apgar score (1 min, 5 min and 10 min), respiratory distress syndrome, bronchopulmonary dysplasia, invasive mechanical ventilation, surfactant use, oxygen supplement, patent ductus arteriosus, thrombocytopenia, blood transfusion, anemia, NSAID use, sepsis
Abdel HA et al., 2012, Egypt [34]	< 32 and > 32	< 1500 and > 1500	low GA, oxygen therapy, frequency of blood transfusions and sepsis
Chen et al., 2011, USA [41]	< 30	< 1500	low GA, Sepsis, oxygen exposure
Hadi and Hamdy, 2013, Egypt [37]	< 32	< 1250	low GA, low BW, Ventilation, blood transfusions, sepsis, Patent ductus arteriosus, IVH
Nair et al., 2001, Oman [36]	< 32	< 1500	low BW, Low GA, TPN

*BW* Birth weight, *GA* Gestational age, *PDA* Patent ductus arteriosus, *CPAP* Continuous positive pressure ventilation, *IVH* Intraventricular hemorrhage, *TPN* Total parenteral nutrition

## Conclusion

Finally, it can be concluded that the present systematic review and meta-analysis summarizes the results of previous studies and provides a comprehensive view of ROP in Iran. Although the prevalence of ROP in Iran is similar to some developing countries, it is much higher than some other countries. Therefore, this fact highlights the importance of preventing and treating ROP and its following complications. To achieve a more favorable level and reduce the prevalence in the coming years, screening and close monitoring by experienced ophthalmologists are essential to diagnose and treat the common complications of prematurity and prevent visual impairment or blindness.

## Abbreviations

CI: Confidence interval
GA: Gestational age
IVH: Intraventricular hemorrhage
PRISMA: Preferred Reporting Items for Systematic Reviews and Meta-Analyses Protocols
RDS: Respiratory Distress Syndrome
ROP: Retinopathy of prematurity
W: Week
